# Parental experiences with their child’s eating disorder treatment journey

**DOI:** 10.1186/s40337-021-00449-x

**Published:** 2021-07-27

**Authors:** Jennifer S. Coelho, Janet Suen, Sheila Marshall, Alex Burns, Pei-Yoong Lam, Josie Geller

**Affiliations:** 1 Provincial Specialized Eating Disorders Program for Children and Adolescents, BC Children’s Hospital, 4500 Oak St., Box 150, Vancouver, BC V6H 3N1 Canada; 2 Department of Psychiatry, University of British Columbia, Vancouver, BC Canada; 3 School of Social Work, University of British Columbia, Vancouver, BC Canada; 4 Division of Adolescent Health & Medicine, Department of Pediatrics, University of British Columbia, Vancouver, BC Canada; 5 Eating Disorders Program, St. Paul’s Hospital, Vancouver, BC Canada

**Keywords:** Pediatric, Eating disorders, Parent perception, Treatment experiences, Family involvement

## Abstract

**Background:**

Parents are integral in the treatment of pediatric eating disorders. The current study was conducted to further understand the barriers and facilitators that parents experience in accessing specialized, tertiary level eating disorder treatment for children and adolescents. The goals of the study were to understand the processes leading to diagnosis and treatment, perceived barriers and facilitators to accessing care, and parents’ experiences over the course of their child’s eating disorder treatment.

**Methods:**

Ten parents whose children were admitted to a Canadian tertiary level specialized pediatric eating disorders program took part in an exit interview upon their child’s completion of treatment in the program. In-depth semi-structured interviews were combined with a visual timeline. Interpretive induction was performed to generate high-level concepts that emerged from the interviews.

**Results:**

Five high-level concepts were identified: (1) delays in identifying eating disorder symptoms, (2) challenges in accessing eating disorder services, (3) the right treatment at the right time, (4) emotional impact on parents, and (5) parental expertise and involvement.

**Conclusions:**

Several barriers were identified by parents that interfered with treatment, including system-related challenges when accessing specialized eating disorder treatment, concerns about a lack of appropriate mental health support for their child, and difficulties with transitioning between community and tertiary level care. Negative emotions, including guilt and self-blame, were common early in the treatment journey. Themes of parental involvement throughout treatment, and parents taking charge of their child’s recovery, emerged across interviews. The results of this study suggest the importance of early identification of eating disorder symptoms, facilitating smoother transitions between levels of care (e.g., community services and hospital-based eating disorder care), and improving clinical decision-making to ensure children and adolescents with eating disorders receive the most appropriate treatment based on their clinical presentation.

**Supplementary Information:**

The online version contains supplementary material available at 10.1186/s40337-021-00449-x.

## Plain English summary

Parents play a central role in pediatric eating disorder treatment. To further understand parents’ experiences over the course of their child’s eating disorder treatment, interviews were conducted with ten parents whose children were admitted to a specialized pediatric eating disorders program. Five major themes emerged across the interviews with parents: delays in identifying eating disorder symptoms, challenges with accessing eating disorders care, the right treatment at the right time, emotional impact on parents, and parental expertise and involvement. Parents shared several barriers that interfered with their child’s treatment, including challenges with transitions between their home community and specialized eating disorder treatment. Parents also commonly spoke about guilt and self-blame when their child was first diagnosed with an eating disorder, though many parents felt relief and improved confidence after being connected with specialized eating disorder treatment. Research is needed to understand how to provide the right treatment at the right time for each child and adolescent with an eating disorder, to ultimately improve clinical care and reduce the barriers experienced by families.

**Supplementary Information:**

The online version contains supplementary material available at 10.1186/s40337-021-00449-x.

## Overview and background

The current study explored the experiences and perceptions of parents whose children received specialized eating disorder treatment. In pediatric eating disorder settings, parents play a central role in their child’s treatment. In Canada and many other countries, family-based treatment (FBT) is generally the first outpatient-based intervention offered to parents whose child has an eating disorder diagnosis, including anorexia nervosa and bulimia nervosa. In FBT, parents learn how to take the lead in their child’s recovery, with the support of the FBT therapist [24]. In higher levels of care, including day treatment and inpatient programs, family involvement is also critical [2]. The central role of parents in eating disorder recovery has empirical support, with guidelines for pediatric eating disorder treatment strongly recommending FBT and highlighting the importance of parental support in higher levels of care [9]. The approach of viewing parents as experts and as key figures in supporting their child’s recovery from an eating disorder is in contrast to early views that parenting failures were one of the causes of eating disorder symptoms, and that eating disorder symptoms were exacerbated with parental contact [14].

Families are viewed as essential in supporting treatment across the developmental spectrum [32]. Yet, guilt and self-blame are common experiences among parents whose child has an eating disorder [16, 40]. Compounding the challenges experienced by parents and caregivers of individuals with an eating disorder, encountering health care providers who are not perceived to be compassionate or understanding can exacerbate the negative experiences of accessing treatment [16]. Furthermore, parents have reported being discouraged from involvement in their child’s care and feeling shut out of treatment for their child [25]. The barriers to families seeking help for their child with an eating disorder therefore appear to include both internal (negative emotions and worries about the role that parents played in the development of the eating disorder) and external factors (negative experiences with health care professionals and treatment programs). A recent systematic review of the literature highlighted several external barriers experienced by families of individuals with eating disorders, which included negative experiences with primary care services, long waitlists, and lack of resources [17].

Despite the reported barriers and challenges in accessing treatment, the literature suggests that families generally are satisfied with specialized eating disorder treatment services once they connect with a treatment team. For example, parents of children admitted to a family-based inpatient treatment program for anorexia nervosa in Norway reported satisfaction with their child’s treatment, with no differences emerging between mothers and fathers [13]. Parental involvement in treatment was appreciated by parents, including the opportunity to stay with their child on the hospital ward and participate in family therapy sessions [12]. Establishing a partnership between the individual with the eating disorder, their family and healthcare professionals is one of the strategies that has been highlighted to facilitate eating disorder treatment [17].

There is a growing body of literature on the experiences of parents of a child with an eating disorder, which highlights the barriers and facilitators to treatment experienced in the pediatric eating disorder system. Parents interviewed about their journey after discovering their child had an eating disorder emphasized challenges in initially finding help for their child, lengthy waits in accessing resources, and strength and determination in supporting their child [4]. Parents have also highlighted that connecting with other parents who have experienced eating disorder services helped them gain a better understanding of eating disorders and hope for the future [26]. Connecting with others with lived experiences may be particularly helpful in the context of families who reported feeling “bewildered” when seeking out resources for eating disorders (p. 5, [26]). Challenges reported by parents also include their interactions with health care professionals in the early stages of seeking support for their child. Parents have reported experiences with health professionals that were demeaning and invalidating, and some parents perceived that the health professionals were reluctant to diagnose anorexia nervosa and did not know how to treat this disorder [40].

### Study objectives

The current study was designed to better understand parents’ experiences in the journey to a tertiary level of care for their child’s eating disorder treatment, and their experiences with treatment in a specialized eating disorders service. Tertiary-level specialized services for eating disorders are indicated for severe eating disorder symptoms that require more support than is available through community services, including medical or psychiatric instability, or failure to make sufficient progress with community supports. Yet, service gaps have been highlighted which can lead to problems in transitioning between different levels of care [39]. The majority of Canadian tertiary eating disorders programs report regular waitlists for services [28], which can lead to further barriers in accessing treatment and navigating the health care system.

To understand parents’ experiences with their children’s eating disorders care, in-depth semi-structured interviews were combined with a visual strategy called a lifeline or timeline. Timelines are used in research to support the collection of information about perceptions of chronological order, importance, meaning of events, and the people and places involved in events [11]. Using timelines together with interviews increases data quality, and is recommended for acquiring comprehensive accounts of an individual’s perceptions and experiences [5]. The goals of the study were to understand parents’ views and experiences about: (1) the processes leading to diagnosis and subsequent treatment for pediatric eating disorders; (2) perceived challenges and supports to accessing and continuing care; and (3) treatment experiences across eating disorder treatment settings, including tertiary level specialized eating disorder treatment.

## Methods

### Participants

Participants in this study were recruited as part of a larger prospective study on treatment outcomes in male and female youths with eating disorders. The recruitment strategy for the prospective study included inviting all male youths who were admitted to the Provincial Specialized Eating Disorders Program for Children and Adolescents at BC Children’s Hospital (based in Vancouver, BC, Canada) to participate, and a sample of matched female participants. At the time of transitioning out of the program, youths and their parents were invited to take part in an exit interview. Parents and youths were invited to complete separate interviews. A total of 25 youths completed the interview. Ten parents of these youths completed the interview, with an equal representation of parents of male and female youths. The current study focuses on data reported by parents. Families were recruited from all program streams. Further details of the levels of services offered in the program at BC Children’s Hospital are reported by Coelho et al. [8]. Details of the demographic and clinical characteristics of participants are provided in Table 1.

**Table 1:** Clinical and Demographic Information about the parent participants and their children

Demographics and Clinical Presentation	Sample Characteristics
Parent and child relationship	Parent 1: Father of a 12-year-old boy Parent 2: Mother of an 18-year-old girl Parent 3: Mother of an 11-year-old boy Parent 4: Mother of a 16-year-old girl Parent 5: Mother of a 14-year-old girl Parent 6: Father of a 14-year-old boy Parent 7: Father of a 17-year-old girl Parent 8: Mother of a 14-year-old girl Parent 9: Father of an 11-year-old boy Parent 10: Mother of a 15-year old girl
Initial treatment of child	Outpatient services: n = 2 Day treatment: n = 4 Inpatient: *n* = 4
Treatments accessed at BC Children’s Hospital Eating Disorders Program	Outpatient + day treatment: *n* = 3 Outpatient + inpatient: n = 3 Day treatment + inpatient: *n* = 2 Inpatient: n = 2
Parents/caregivers living with child	Both parents: *n* = 7 1 parent and partner/step-parent: n = 2 Mother: *n* = 1
Age of child (at time of interview) Age of parent	*M* = 14.64 years (*SD* = 2.49) *M* = 44.8 years (SD = 5.07)^+^
% median Body Mass Index (mBMI) Start of treatment End of treatment	*M* = 84.94 (*SD* = 9.06) *M* = 96.67 (*SD* = 11.45)
Duration of tertiary-level treatment	*M* = 27.31 months (*SD* = 18.99) Range = 7.83–67.25 months

+ Parent age was available for only 5 of the 10 parent participants, as only some parents participated in the larger prospective study and completed measures including a demographic questionnaire

In the current study, there was a focus on parents of children with restrictive eating disorders. The majority of youths (*n* = 8) had a diagnosis of anorexia nervosa – restricting type (AN-R), while two youths had a diagnosis of avoidant restrictive food intake disorder (ARFID). Diagnoses were recorded from medical records, which were assigned by a psychiatrist or doctoral-level psychologist with specialized experience in eating disorders.

### Procedure

Parents were interviewed individually by a member of the research team. A semi-structured interview was employed, using an adapted life history methodology [11] in order for parents to provide accounts of their experiences over time. This type of interview is useful for understanding how sequences of events are perceived and is particularly helpful for investigating transitions. Parents were asked to draw a timeline of the significant events or milestones in their child’s eating disorder treatment journey, starting from the time parents perceived that their child first started to change their eating behaviour until their child’s transition out of the BC Children’s Hospital Eating Disorders Program. Details about the interview methodology and script are available in Supplementary Materials.

All interviews were audio recorded, and transcribed verbatim by a professional transcription service. Interviews ranged from 20 to 86 min in length (*M* = 39 min, SD = 19). Parents were offered compensation for parking expenses (if applicable) as part of their participation in this study, and youths and their parents who completed the interview were offered a gift card for the family with a value of $10.

Organization of the data and analysis of the interviews followed a process of interpretive induction [19]. This analytic approach was used to delve into the experiences and perceptions communicated by parents. Interpretive induction recognizes that the researchers bring their own understandings of participants’ experiences to the analysis. This is important in this study given the extensive experience of the team leader (JC) in working with families in eating disorder programs. The other team members who led the analyses (JS and SM) brought their experience of research with families to the data analysis.

To start the analytic process, two members of the research team (JC and JS) reviewed all interview transcripts and timelines together, in consultation with another research team member (SM). Initially, the significant milestones and experiences identified by participants were identified and extracted from the transcripts and timelines. The central themes that emerged across participants’ interviews were summarized, with concepts that were common across several participants classified as central themes. Outliers, or treatment experiences that were not common across participants were also recorded. Differences in the key themes identified by the members of the research team leading the analysis (JC and JS) were reviewed and resolved for each participant. Similarities across participants’ experiences were then identified and used to create conceptual categories. Finally, the data were coded into high-level concepts based on grouping of the main conceptual categories that emerged.

Finally, an individual with lived experience (a parent whose child had previously received treatment in the Eating Disorders Program at BC Children’s Hospital, who was not part of the participant group) engaged in a review of the analysis. The parent representative provided feedback about the themes and summary of results, to improve the credibility and reliability of the results. This feedback led to a clarification of the high-level concepts, and led to a more balanced focus on milestones identified by parents across their child’s treatment journey (i.e., experiences connected with both entry into and transitioning out of tertiary care).

## Results

**Fig. 1:**
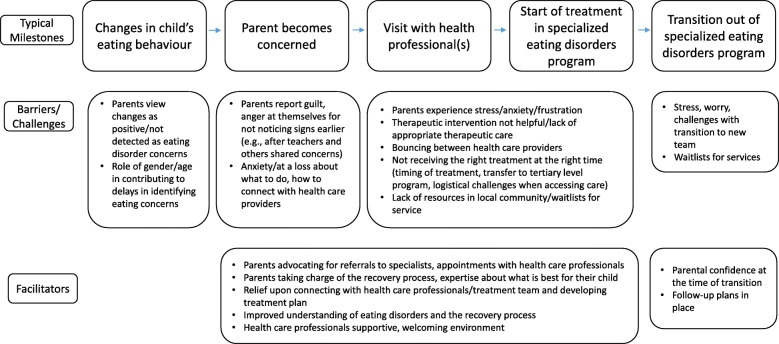
Representation of milestones that emerged in parent interviews, with conceptual categories relating to challenges and facilitators to accessing appropriate care

Five high-level concepts emerged: (1) delays in identifying eating disorder symptoms, (2) challenges in accessing eating disorder services, (3) the right treatment at the right time, (4) emotional impact on parents, and (5) parental expertise and involvement (see Table 2 for details of conceptual categories and high-level concepts). In developing the conceptual categories and themes, the pattern of milestones was examined for each participant. It is noteworthy that there was no common pathway to tertiary level specialized eating disorder treatment. The majority of parents (eight of the ten participants) described hospitalization for medical stabilization preceding their child’s start of treatment in the specialized eating disorders program. Yet, within the pathway for those who were hospitalized, there was diversity in the contacts that families had prior to hospitalization. It is also noteworthy that all parents reported experiencing challenges at some point during their child’s treatment journey. Three parents reported having an assessment and starting treatment at BC Children’s Hospital without barriers to the referral process or bounces through different health care professionals. Yet, the emotional toll on parents in the early stages of the eating disorder was a common consideration. Figure 1 depicts an overview of the typical key milestones reported by parents, with conceptual categories relating to challenges and facilitators that arose. The high-level concepts that emerged across the conceptual categories are detailed below.

**Table 2:** Summary of the conceptual categories that emerged from parent interviews, and the classification of these categories into high-level concepts

High-level Concepts	Conceptual Categories
Delays in identifying eating disorder symptoms	• Parents did not recognize eating disorder symptoms or were not concerned in the early stages of changes to eating behaviour • Eating disorder slipped under the radar due to gender/age
Challenges with accessing eating disorder services	• Delays in referral to appropriate services • Lack of appropriate services in home community • Delays in accessing treatment/waitlists
Right treatment at the right time	• Lack of therapeutic care or unhelpful therapeutic care • Bouncing between care providers • Timing of admission/logistical issues affecting care
Emotional impact on parents	• Anxiety about accessing an appropriate treatment, feeling at a loss about how to best support their child • Anger, frustration, desperation and guilt in the early stages of the treatment journey • Relief upon being connected with an appropriate treatment
Parental expertise and involvement	• Caregiver knows best • Advocacy in treatment planning • Developing a better understanding of the recovery process • Involvement during treatment helped improve confidence at the time of discharge

### Delays in identifying eating disorder symptoms

Parents highlighted a gap between first noticing changes in eating behavior symptoms and being concerned and connecting with health professionals. Several parents indicated that they did not initially recognize eating disorder symptoms, or were not initially concerned about changes (e.g., perceiving that their child was increasing fitness, focusing on healthy eating, or going through a period of being a “picky” eater). Parent 2 shared the challenges in detecting changes that were gradual, making it difficult to notice the changes. Furthermore, these changes were in the context of her child growing taller, leading to an initial attribution of the changes in body shape to a growth spurt. Some parents shared that they had initially not been concerned with some of the eating-related changes they noticed their child making, but when others in their community (e.g., teachers, dance instructors, friends) raised concerns they started reaching out to health professionals.

Four of the five parents of boys highlighted gender as potentially contributing to delays in recognizing eating disorder symptoms. One father (Parent 9) suggested that age and gender contributed to delays in linking his child’s behavior with an eating disorder, indicating that “*we didn’t even think of anorexia.* […] *it just wasn’t on our radar, and he was a 10-year-old boy*”. Another father (Parent 6) shared that “*they’d diagnosed him with anorexia.* […] *And then—so it was like, holy—okay, what is this?* […] *And I’d never heard about it with guys, right?*”

### Challenges in accessing eating disorder services

Several parents experienced barriers after recognizing eating disorder symptoms in their child, and shared their impressions that health professionals that they visited (e.g., family doctors) were not taking their concerns seriously. A mother of a 14-year-old girl (Parent 5) shared her experience with expressing concerns about her child’s eating to a family doctor, and being re-assured and advised to return one month later. In the meantime, the parent continued to feel that something was wrong and sought out support from a second doctor, who advised her to bring her daughter to the emergency room, leading to a hospital admission for medical stabilization. Similarly, Parent 1 shared the rapid onset of medical instability after his son’s initial changes in eating behaviour:

*Um, it happened in a—in a very short space of time.* […] —*he started to eat more healthily because he wanted to get fitter to*—*primarily, to play soccer. So we thought, “Okay, that’s a good thing.”* […] *Um, and it just seemed to accelerate and snowball from there initially.*

Parent 1 explained that he took his child to the doctor and was told “*he’s okay.”* However, like Parent 5, he continued to have concerns over the next days, and took his child to the emergency department, and his child was admitted for medical stabilization.

System-related barriers, including a lack of resources in non-urban communities, and waitlists for services also emerged. For families outside of urban centres, limited resources and a lack of experience of local health professionals with pediatric eating disorders were barriers to accessing appropriate treatment.

Parent 7: *And the trouble with us and the most frustrating part with us is that there are zero resources for this in* [family’s home community]. *There is nothing for child and youth mental health up there. There is no one for eating disorders. Um, so that was a frustrating struggle to get the ball rolling in the first place.*

In contrast, other families who lived in urban and suburban areas had eating disorders programs in their home community, yet parents described facing waitlists for these services.

Parent 10: *Uh, so I called* [local community] *Eating Disorder Program. They said they’re full, she’s on a waiting list. I said I cannot wait, and what—what should I do? And they said bring her to emergency. I said, ‘What are they going to do in emergency? They’re not going to help me, like, psychologically.’*

Parents reported encountering waitlists across the treatment journey, including at assessment and seeking initial connection with eating disorder specialists, and at the time of transfer from regional services to specialized provincial services (e.g., from hospital in the family’s local community to the specialized pediatric hospital). Waitlists were also reported at the end of treatment, as families transitioned out of the tertiary care program. For some families who transitioned from the tertiary program to secondary level community eating disorders programs for follow-up, the tertiary program offered bridging services. Bridging services allowed families to continue to attend outpatient appointments and medical monitoring at the tertiary site while on the waitlist for services in their local community program.

### The right treatment at the right time

Across interviews, parents highlighted bouncing between care providers and treatment services, a lack of psychological care/unhelpful therapeutic interventions, logistical challenges when accessing care, and less than optimal timing of intensive treatment. These challenges related to the concept of finding the right treatment at the right time for their child.

Several families reported experience with multiple health care professionals and teams during their child’s treatment journey prior to starting treatment in the specialized pediatric eating disorders program. Challenges arose for individuals in rural or remote communities while receiving treatment in their home regions and waiting for transfer to specialized care. Parent 3 described her child’s deterioration in the local hospital while waiting for transfer, indicating that it felt like the delay in accessing specialized care was “*setting him up for failure* [*…*] *So we just sat there and waited and waited.* [*…*] *Yeah, as things got worse and worse and worse [laugh]. Yeah.”* Parent 3 highlighted a lack of communication, and miscommunication across team members within the community hospital, as well as between community and tertiary care, that was associated with delays and challenges. Several parents also shared challenges with navigating how to access appropriate specialized pediatric services. Parent 2 described her thoughts about determining how to access specialized eating disorder services: *“I needed somebody to help me provide a plan of attack, how was I going to go about helping my son?”*

Bouncing between treatment services and care providers also occurred for families whose child was admitted to the specialized pediatric hospital, between medical hospitalization, mental health teams, and different teams within the eating disorders program (outpatient, day treatment, and inpatient services). Part of the frustration raised by parents about the bounces between care providers related to a lack of continuity in care providers, with rotation through health professionals.

Parent 1*: “I guess the system, the way that system is designed isn’t a good thing for a kid with eating disorders. He needs continuity throughout his care here, I think* […]*”*

Parents also described a division between medical and psychological care, with initial treatment focused only on medical stabilization:

Parent 10: *We were there to stabilize medically, and that’s what they were basically doing. You know, diet, high calorie diet. She was monitored 24/7. She was not able to leave the bed, right? They were very strict, but nothing like mentally that was done for her.*

Parents commonly raised their desire for more mental supports for their children during the treatment process. Yet, several parents highlighted the importance of timing when offering mental health supports to children who were malnourished or medically unstable. For example, parents whose children had accessed mental health supports in the early stages of the eating disorder indicated that the individual treatment that was initially offered to their child was not a good fit:

Parent 9: *Um, so we went to a counsellor for a couple of sessions. It was not useful.* [*…*] *I think the reason that—you know, when you’re malnourished and your brain isn’t working properly, you can’t—counselling just doesn’t work.*

Logistical, staffing issues, and challenges with communication between health care professionals also led to barriers in the treatment processes. For some families, staff holidays and reduced programming (e.g., over the summer and December holiday periods) led to delays and gaps in services, and longer waits to see specialists. Challenges also occurred with communication across health professionals. One parent spoke about the long process in working towards a process of communication and ensuring all members of the inpatient treatment team were on the same page as parents, leading to challenges that were perceived to have a significant impact on the treatment process:

Parent 9: *Um, and so it’s how that information gets communicated down for each patient. So eventually, like I said, everyone got it and it was fine, but it took, you know, a month, um, which is [laugh] a long time.*

Seven of the ten participants experienced their child stepping up to more intensive services (e.g., outpatient to day treatment, or day treatment to inpatient) during their treatment in the program. This step up to more intensive treatment was perceived to be helpful; however, four parents indicated that a more intensive treatment option would have been helpful earlier on. Concerns about the timing of transitions to more intensive treatment also emerged in the interviews. A parent who had been attending outpatient services for an extended time with her child described her advocacy for a step-up to the day treatment program.

Parent 8: *I said, ‘This is—none of this is working, right? She’s—I think we need to go to the next level here. We need to go to the next step.’*

This parent also suggested that starting a more intensive treatment earlier could have been helpful, while recognizing that recovery is a process:

*[laugh] it was like, maybe we could have done something, um, you know, earlier in terms of putting her into a—into it* [day treatment]. *But—but again, you know, sometimes you just have to walk the road, right?*

Parent 1 summarized the challenges his family experienced in finding the right treatment at the right time for his son:

*But the mental side of things where obviously he did need help, was hopeless really, and—and that was very frustrating. And—and again, not—now, I get it’s not that easy. I know it’s easy for me to see it afterwards and say, “Oh, you know, if we could have done this or done that,” but—but maybe there’s a better way* [*…*] *to try and get the right fit for the right person to the right patient I guess is what I’m saying really*.

Despite the challenges families described, all parents highlighted their positive experiences with the treatment team in the tertiary eating disorders program.

### Emotional impact on parents

Many parents shared the negative emotions they experienced during the early stages of identifying the eating problem and seeking out treatment for their child. Parents described feeling guilty, as well as angry at themselves for not identifying the eating problem earlier, or losing time in getting connected with appropriate services. Parent 2 shared: “*And, [sighs] I guess there was anger at myself too for not seeing the signs earlier or accepting the signs earlier I guess.”* Anxiety, stress, and feeling unsure about how to best proceed was also common across interviews. One parent shared the strong emotions she experienced after initially trying to connect with health professionals about how to support her daughter in the context of continued weight loss (Parent 10): “*And then, um, I was—like, I was desperate. I didn’t know what to do with her.”* Similarly, parents spoke about the challenges with getting connected with care providers. Parent 9 shared*:*

*Um, so anyway, we did try the mental health route before, but that was originally one of the most difficult things. I had no idea how to reach into the mental health world, um, you know, how to get support, how to get access.*

Parents commonly noted anxiety, frustration, and feeling at a loss about how to proceed in the early stages of accessing care for their child, suggesting challenges in the transitions between services in the community and specialized eating disorder care.

There was a division in experiences after parents connected with specialized eating disorder services. Some parents described significant relief upon connection with the specialized eating disorders program. Parent 3 described her emotional experience upon her son’s admission to the inpatient eating disorders program:

*Relief. Yeah, relief that we were where we needed to be and just hearing from other people that have been to the eating disorder clinic from* [the family’s home community] *and how amazing it is and how much they help* […]

In contrast, some parents described frustration throughout the treatment process. Parent 4 described her experiences during her daughter’s treatment in the specialized program, sharing her impression that further medical investigations and psychiatric interventions would be needed to support her child’s recovery, and feeling that parents were not on the same page as the health care team:

*Well, certainly [pause] you know, disappointed and, again, helpless* [*…*] *But um—so yeah, I still—I’m still beating my head against a wall* [*…*]

Setbacks after initiating treatment for the eating problem were also frustrating to parents., Parent 7 spoke about the slow process of change and on-going eating disorder symptoms leading to frustration during his daughter’s admission to the inpatient program:

*I—at the time, I was frustrated because um, I was wondering why the thinking wasn’t changing, right. Why—I mean, she’s here being treated. Um, why isn’t there enough being done on the mental um, you know, the—the cognitive uh, shift?*

One father recognized the need to manage his emotional state, in order to best support his child’s recovery, and described the process of adapting during his child’s treatment in the eating disorders program:

Parent 6: *But I mean, it’s stressful, but you know you’d—you’d do anything for your kids, right? So you just adapt. It’s like when they were—when they were born, you don’t get any sleep, but you adapt, right? So you just—I just adapted to the situation and just kind of—okay, I’m going to get through it.* [*…*] *So I try not to be too emotional about it ‘cause I have to be the strong one for him. And so the thing is, if I’m falling apart, then it’s going to be no use to him.*

For the majority of parents, the negative emotional state that was typical in the early stages of the treatment journey shifted to feelings of confidence and hope at the time of transition out of the tertiary eating disorders program. Parent 9 shared that both parents had been leading their son’s care over the course of inpatient treatment, which helped to bolster the family’s confidence about the upcoming transition back to their local community:

*Well, I feel like for a long time, we’ve been the main supports.* […] *so I think we’ve been feeling quite confident for a long time about the home piece.*

Similarly, Parent 2 described her positive emotional experience that was associated with her son’s progress in treatment:

*Elated. [laughs] Elated because he had reached um, well within what was acceptable* [weight] *range for his height and age. So I was very pleased that, uh, that he got there so quickly*. […] *Like, it was, it was monumental.*

This parent also recognized recovery as an on-going process, describing her feelings at the time that her child transitioned out of the tertiary level program:

*A lot more confidence. A lot more at ease about it. Uh, I know that there’s still a ways to go. But I think we’re on the right track, and, you know, he’s at a, he’s at a good place.*

However, a subset of parents spoke about the challenges of leaving a team that was familiar with their child’s history and the uncertainty of some of the follow-up options in their local community leading to worries and fears. Parent 4 described the mixed emotions associated with her daughter’s upcoming transition out of the tertiary program: “*Um, I think we’re all a little bit scared and worried but, you know, hopeful that she will do well.”*

### Parental involvement and expertise

As a program offering family-based treatment to outpatients and centered around family involvement for higher levels of care (day treatment and inpatient eating disorders services), all parents were engaged in a collaborative treatment process from the time of assessment through to the completion of treatment in the tertiary program. The health care professionals in the program worked with children and parents to develop an individualized care plan that incorporated parental expertise of their child. Several parents expressed concerns about certain elements of the treatment program, and worked collaboratively with the team to mitigate their concerns, which facilitated positive treatment experiences.

Parents also described gaining confidence and skills throughout their child’s treatment, with the support of the team and skills groups offered to parents (Parent 5): “*That’s a great thing that started helping, just trust my—you know, my own decisions.”* In the context of the negative emotional impact of eating disorder treatment on parents, one parent described feeling helpless at times, particularly with supporting her child in the context of other family stressors, while at the same time recognizing *“I’m just, you know, a mother that’s never gonna give up on her.”* (Parent 4).

Parental involvement throughout treatment, and parents taking charge of the recovery process also emerged across interviews. The family-based therapy approach involves parents taking charge of their child’s eating, and asking parents “to be “*directly responsible* for changing eating-related behaviors at home” ([22], p. 138). Across treatment modalities that families received in the program, parents described finding ways to take charge and support their child’s recovery. One parent spoke about her thoughts when starting FBT:

Parent 10: *I was hoping that she would be admitted here or, like, something more intense done for her. Uh, so I was pretty sceptic first when it started, and then actually* [*…*] *I don’t know when exactly—I noticed that actually I’m learning a lot from this to manage* […]

Parents also described their firm stance in decision-making during their child’s treatment. One mother shared her approach in planning for her daughter’s step up to day treatment from outpatient care.

Parent 8: *She was quite resistant* [about day treatment]*. She was crying and she didn’t want to, right? And she was afraid. She was frightened of the whole idea about it. And, um—but I was, uh, pretty strong about it and pretty adamant that that’s what has to happen, and there really—this is not a debate*. *[laugh]*

This parent went on to describe her firm attitude in supporting her child’s eating at home, and continuing with rules and expectations about eating that were established during the day treatment program:

[…] *you know, these 14 year olds, 15 year olds, you know, they just want to be the boss. It’s going to be their way. And you have to recognize, no, you’re the parent, and they’re the child, right? [laugh] And this is how it’s going to be.*

Finally, parents described improved understanding about the recovery process as their child’s treatment progressed. Several parents shared their shift from expecting a focus on psychological support for their child in the early stages of treatment, to understanding that weight restoration and normalization of eating are necessary prior to changes in their child’s psychological well-being. For example, Parent 6 described:

*Um, but I understand now um, that that’s—you know, you can’t start that until the body’s healthy. Body has to get healthy first. Brain has to get healthy before that kind of treatment can start. Otherwise, you know, it’s potentially a waste of time and can be detrimental even too.*

Parents also spoke about their change in behavior over the course of treatment to support their child’s recovery, as they learned more about eating disorders and how to be supportive and navigate triggers for their child. Parent 9 reported:

*… there’s all kinds of pitfalls with this disease. You know, like, I learnt early on that you can’t say, ‘You know, I really think you look a lot better.’ That’s a bad thing to say. [laugh]* […] *Um, so you know we choose our words now around height in particular, that you’re growing upwards, and you need to support that growth for growing taller and growing stronger”*

## Discussion

The five high-level concepts that emerged from interviews with parents emphasized the challenges and facilitators that families have experienced in the process of accessing and receiving specialized eating disorder treatment in a Canadian tertiary care setting. These concepts included delays in identifying eating disorder symptoms, challenges with accessing services for eating disorders, the right treatment at the right time, the emotional impact on parents as their child’s treatment plan was implemented, and parental involvement and expertise. The specialized eating disorders program is based in a pediatric hospital that offers patient and family centered care [3]. This approach includes empowering families to care for their children, and collaborating with children and families as partners in treatment. Consequently, all parents were engaged with the treatment team throughout the treatment process, and several parents highlighted how their involvement facilitated treatment and helped prepare for their child’s completion of treatment in the program.

Collaborative partnerships between the individuals with the eating disorder, their family and healthcare professionals facilitate eating disorder treatment [17]. This partnership may also improve transitions between community and tertiary treatment (both when entering the tertiary program and upon transition back to the community). Parental engagement and collaboration differed markedly from earlier reports of parents being discouraged from involvement in treatment [25]. The emergence of the theme of parental expertise and involvement in their child’s treatment mirrors themes that emerged in a study of clinicians working with pediatric eating disorders, in which parental empowerment was viewed by clinicians as essential to support a child’s recovery from anorexia nervosa [10].

Many parents shared stories of delays in making the first connection with a health professional, as they did not notice the severity of the changes that were happening with their child’s eating. In contrast, some parents shared negative experiences with the professionals that they first encountered, reporting their impression that the doctors were not taking the concerns seriously, or did not have timely follow-up. Negative experiences with primary care professionals have been previously reported, with recommendations that training and support for primary care professionals can help improve the experience for patients and families, and better utilize the continuum of services [17]. It is also noteworthy that the negative experience reported with health care professionals was not universal, and some parents noted timely access to care with minimal barriers.

The reflections regarding the timing of treatment suggested that a subset of parents would have preferred a more intensive treatment to be offered sooner in their child’s treatment. Of the parents whose child stepped up to a more intensive treatment, there was a division between parents’ perceptions. Some parents did not comment on the timeline or process of the step up, while others reported that this step up would have been helpful earlier in the treatment. Recent Canadian guidelines for pediatric eating disorders strongly recommend that the least intensive treatment environment be provided for children and adolescents, particularly for those with a recent onset of eating disorder symptoms [9]. Yet, Couturier et al. [9] note that research evidence is not yet sufficient to guide how to optimize the level of care. Given that more than half of the families in the current study who experienced a step up to more intensive treatment reported that they believe this would have been helpful earlier, the development of indicators of the need for re-evaluating level of treatment would be helpful. There is emerging evidence for adjunctive interventions for individuals who are not responding adequately to treatment. An adaptive treatment in which intensive parental coaching is added to FBT has been developed [23]. Development and adoption of clear criteria for clinicians to consider in the early stages of treatment, including those who start treatment at a higher level of care such as day treatment, would help clinical decision making regarding the most appropriate treatments based on clinical presentation of children and youths with eating disorders.

The emotional impact on parents was overwhelmingly negative in the early milestones reported by parents, with reports of guilt and self-anger, and feeling at a loss about how to proceed. This negative emotional impact paralleled the emotions that emerged in a meta-synthesis of the literature, in which parents reporting feeling confused, frustrated, guilty and powerless to help their child [33]. Similarly, parents based in Canada commonly reported guilt and shame upon discovering that their child had an eating disorder [40]. The negative emotional impact reported in the current study tended to shift to positive emotions as treatment progressed, and appeared to be connected with an improved understanding of the treatment process, and working collaboratively with health care teams.

Several parents reported their perception that there was a lack of support for mental health during treatment. This finding mirrors previous parent reports of experiences with medical stabilization for anorexia nervosa, in which 40% of parents reported a desire for their child to have mental health services during hospitalization [6]. Similarly, past studies report that parents perceive that treatment focuses primarily on the physical, as opposed to psychological, health of their child [33]. Some parents noted that they learned that offering mental health treatment while their child is in a malnourished state could be unhelpful or even detrimental. However, there is emerging evidence indicating that mental health supports that focus on engagement can be helpful, even for individuals who are malnourished. For example, cognitive remediation therapy (CRT) is an approach that can be helpful even for children and adolescents with severe eating disorders who are significantly underweight. CRT is a brief intervention that targets thinking styles, including cognitive flexibility and detail-focused thinking [36]. CRT is feasible and has been used across different treatment settings, including medical stabilization [38], FBT [21], and inpatient treatment [15]. CRT represents a promising adjunctive treatment that is compatible with an initial focus on physical recovery [9]. It may also be helpful for parents to understand the non-specific supportive care that is offered by multi-disciplinary team members, including nursing staff, that is part of mental health support in eating disorder treatment settings [31].

Parents generally saw transition out of the tertiary treatment program as one of the steps in their child’s treatment journey, with all families planning follow-up care for medical monitoring at a minimum and most families connected with follow-up therapy for their child’s eating disorder in their local community. Parents of youths with an eating disorder have highlighted the lengthiness of the recovery process [35]. In a survey of parents of youths who had a diagnosis of an eating disorder, only 20% reported that their child had completely recovered, and reported that physical recovery preceded cognitive recovery [1]. Treatment with a focus on improving quality of life has been recommended as a follow-up support, after reduction of eating disorder symptoms has been achieved [1].

One of the themes that emerged in the current study were difficulties with transitioning between community and tertiary level services. These challenges arose for families both in the early stages of their child’s treatment journey, when parents were navigating the supports available and trying to work with health professionals to develop a treatment plan for their child, as well as at the end of tertiary care services, when families were returning to services in their home community. Challenges in navigating transitions between levels of service have been previously reported in the literature [39]. Demographic factors and the geographic location of families seeking treatment may be associated with the barriers to accessing appropriate care. Males and those from non-affluent backgrounds are less likely to receive eating disorder treatment [34]. Furthermore, males, those with socioeconomic disadvantages, and those from rural and remote areas had an increased risk of being readmitted to hospital, which was associated with lack of access to appropriate community mental health services [20]. The current study provided some further evidence for the risk males face in delayed access to treatment, with the majority of parents of boys indicating that there were delays in identifying their child’s eating-related concerns as an eating disorder. Assessing whether services are equitable and improving navigation and access to services in home communities have been identified as research priorities as part of the Canadian Eating Disorder Priority Setting Partnership [29].

### Limitations

Limitations of the study include the small sample size of parents in the current study. However, this limited sample size is representative of qualitative studies on parents who have a child with an eating disorder, with 6 of the 10 studies included in a recent metasynthesis of the literature comprising similar sample sizes of 9–14 parents (see [33]). Furthermore, parents had experience with a variety of treatment intensities, including outpatient, day treatment and inpatient services. Given that the majority of youths stepped up to a higher level of care during treatment, the diversity of treatment settings that parents had reflects the fluid nature of the treatment streams in the eating disorders program, with some youths moving in and out of more intensive services or hospital admissions as needed. However, the diversity of treatment experiences represented by the small sample of parents may have limited the commonalities that emerged during the interviews. The parents who participated in this study also all were supporting a child with a restrictive eating disorder. The results therefore may not generalize to other eating disorder symptom presentations.

Two members of the research team also had clinical roles in the eating disorder treatment program (JSC and PYL), leading to the potential for bias in the interpretation of results. This potential for bias was balanced by the inclusion of two non-clinical members as part of the core analysis team. There was also a potential for bias in the themes raised by parents who responded to the invitation, as only 40% of parents of the 25 youths interviewed agreed to take part in the interview. The recruitment method in which all males and a selection of matched females admitted to the treatment program were invited to participate in the study also led to a higher proportion of parents of males in the current study relative to the proportion of males that is typically seen in tertiary treatment settings. This equal representation is a strength of the study, given the marginalization of males in eating disorder research [27]. There may be some gender differences in the experience of tertiary treatment services based on gender. One such theme emerged in the current study, in which the majority of boys’ parents highlighted gender as contributing to delays in identifying eating disorder symptoms. However, no further gender-specific conceptual categories emerged in the analyses.

### Future directions

This study demonstrated that many families experienced delays in initially identifying an eating disorder or accessing supports for their child’s eating and weight concerns. These findings suggest the importance of primary care professionals in helping families to identify clinically significant eating-related concerns, and helping to navigate referrals to appropriate services, which has been recommended by Johns et al. [17]. Initial evidence for knowledge exchange partnerships between Canadian tertiary care and primary care services for eating disorders has been published, which suggests that patients of primary care clinicians who were part of this partnership had good outcomes [37]. Although this program included supplemental training for clinicians who worked with pediatric populations, patient outcome variables were reported only for adults [37]. Therefore, future research investigating the impact of knowledge exchange partnerships in facilitating health care services for pediatric eating disorders is needed.

One of the conceptual categories that arose in the current study was parental guilt, which is common with previous reports [16, 40]. Parental guilt has been attributed in part to parental explanations about the cause of their child’s illness, which for some parents are based on worries about having passed on “bad genes” while for others are attributed to parenting practice or other environmental factors [30]. Psychiatric genetic counselling leads to improvements in parental self-efficacy and empowerment [18]. Genetic counselling is not dependent on genetic testing, and has been recommended as an approach that can help families make sense of the complex role of genetics and environment in eating disorders [7]. Future research examining the impact of psychiatric genetic counselling on parental guilt and self-blame during the early stages of eating disorder diagnosis and treatment is warranted.

## Conclusions

The five high-level concepts that emerged from interviews with parents were delays in identifying eating disorder symptoms, challenges in accessing eating disorder services, accessing the right treatment at the right time, the positive and negative emotional impact on parents, and parental involvement and expertise. Several barriers were identified by parents that interfered with treatment, including system-related challenges when accessing specialized eating disorder treatment, concerns about a lack of mental health support for their child, and difficulties with transitions between community and tertiary level care (both when entering and exiting tertiary treatment). Negative emotions, including guilt and self-blame, were common early in the treatment journey, though parents generally reported higher confidence as treatment progressed. Strengths of the study include the equal representation of parents of girls and boys, the focus on families who accessed specialized tertiary-level care, and the interview methodology that allowed for an in-depth exploration of parental perceptions of the treatment journey. The results of this study suggest the importance of early identification of eating disorder symptoms, facilitating smoother transitions between levels of care, and improving clinical decision-making to ensure children and adolescents with eating disorders receive the most appropriate treatment based on their clinical presentation.

## Supplementary Information

**Additional file 1.** Interview script and procedure.

## Data Availability

The datasets generated and/or analysed during the current study are not publicly available, to protect the confidentiality of participants as details provided in the interviews could be personally identifying. The de-identified aggregate results that were generated based on the interviews are available from the corresponding author on reasonable request.
