# Correction to: Parental experiences with their child’s eating disorder treatment journey

**DOI:** 10.1186/s40337-022-00625-7

**Published:** 2022-07-19

**Authors:** Jennifer S. Coelho, Janet Suen, Sheila Marshall, Alex Burns, Pei-Yoong Lam, Josie Geller

**Affiliations:** 1 Provincial Specialized Eating Disorders Program for Children and Adolescents, BC Children’s Hospital, 4500 Oak St., Box 150, Vancouver, BC V6H 3N1 Canada; 2 Department of Psychiatry, University of British Columbia, Vancouver, BC Canada; 3 School of Social Work, University of British Columbia, Vancouver, BC Canada; 4 Division of Adolescent Health and Medicine, Department of Pediatrics, University of British Columbia, Vancouver, BC Canada; 5 Eating Disorders Program, St. Paul’s Hospital, Vancouver, BC Canada

## Correction to: Journal of Eating Disorders (2021) 9:92 10.1186/s40337-021-00449-x

Unfortunately, the original version of this article [1] contained two errors in Table 1:

1. The duration of tertiary-level treatment listed in Table 1 should read: M = 10.77 months (SD = 7.49). Range = 3.09–26.53 months.

2. The parent and child relationship for parent 2 listed in Table 1 should read: Mother of an 18-year old boy.

The revised Table 1 is included in this Correction. The original article has been corrected accordingly.

**Table 1:** Clinical and Demographic Information about the parent participants and their children

Demographics and clinical presentation	Sample characteristics
Parent and child relationship	Parent 1: Father of a 12-year-old boy Parent 2: Mother of an 18-year-old boy Parent 3: Mother of an 11-year-old boy Parent 4: Mother of a 16-year-old girl Parent 5: Mother of a 14-year-old girl Parent 6: Father of a 14-year-old boy Parent 7: Father of a 17-year-old girl Parent 8: Mother of a 14-year-old girl Parent 9: Father of an 11-year-old boy Parent 10: Mother of a 15-year old girl
Initial treatment of child	Outpatient services: n = 2 Day treatment: n = 4 Inpatient: n = 4
Treatments accessed at BC Children’s Hospital Eating Disorders Program	Outpatient + day treatment: n = 3 Outpatient + inpatient: n = 3 Day treatment + inpatient: n = 2 Inpatient: n = 2
Parents/caregivers living with child	Both parents: n = 7 1 parent and partner/step-parent: n = 2 Mother: n = 1
Age of child (at time of interview) Age of parent	*M* = 14.64 years (*SD* = 2.49) *M* = 44.8 years (SD = 5.07)^+^
% median Body Mass Index (mBMI)	
Start of treatment	*M* = 84.94 (*SD* = 9.06)
End of treatment	*M* = 96.67 (*SD* = 11.45)
Duration of tertiary-level treatment	*M* = 10.77 months (*SD* = 7.49) Range = 3.09–26.53 months

^+^Parent age was available for only 5 of the 10 parent participants, as only some parents participated in the larger prospective study and completed measures including a demographic questionnaire
