# Supplementary material for: Parental experiences with their child’s eating disorder treatment journey
Source: J Eat Disord. 2021 Jul 27;9:92. doi: 10.1186/s40337-021-00449-x (PMC8314586; doi:10.1186/s40337-021-00449-x)
Supplement: Supplementary file 1 — Additional file 1. Interview script and procedure. [file 40337_2021_449_MOESM1_ESM.docx]

Supplementary Material:

Interview script and procedure

Parents were provided with a large piece of paper and markers, and were asked to represent each milestone/event with a symbol, picture, or a few words. Parents were invited to complete the lifeline picture and then to describe the events to the research assistant, or to verbally describe the events as they drew the lifeline. For each event identified, parents were prompted to describe their thoughts and feelings associated with the event. They were also prompted to ensure that all key events leading up to treatment were included in the lifetime (e.g., process of their child’s diagnosis of an eating disorder, referral to an eating disorders program, and admissions to eating disorder treatment services). Parents were asked about their experiences during treatment at BC Children’s Hospital (e.g., staff support, suggestions for changes to the program). Finally, parents were asked about the follow-up care that was planned for their child, and their perceptions of the care available to their family after discharge.

The interviews assist in identifying:

- Processes leading to diagnosis and treatment.
- Perceived barriers to accessing and continuing care.
- Perceived supports for accessing and continuing care.
- Experiences of the response of health professionals and the ED treatment setting.

Script:

Thank you so much for agreeing to participate in the interview portion of this research project. Thank you also for completing the questionnaires.

As we mentioned in the consent forms, I will be audio-taping this interview to make sure we have accurate records of what is going on. I’ll just turn on the recorder now.

*** turn on the audio-recorder ***

“We would like to get a sense of what led up to you entering treatment here in the Eating Disorders Program – to do this, we are going to ask you to draw a timeline on this piece of paper. Use a graph or traveling line to express your feelings in relation to events and milestones in your life. Your lifeline is to start from the time that you first started to change your eating behaviour and continue to present, beginning from the left end of the paper and moving to the right. Each event and milestone is to be labeled by a symbol or picture image.”

After the participant completes the timeline, the interviewer will review the key events, from left to right, asking for each event:

What are some of the thoughts went through your head during this event [stating name of event, e.g., assessment at doctor’s office, starting treatment]?

*Add “tell me more about this” if required or to clarify*

How did you feel during this time?

*Add “tell me more about this” if required or to clarify*

After completing this process, the interviewer will ensure that keys events leading up to treatment are including on timeline, including:

- Process of diagnosis/referral, and time elapsed between the first changes in eating behaviour and receiving first diagnosis/referral for treatment.
- Current treatment, and any preceding treatment here or from primary or secondary service providers prior to current admission.
- Interviewer will inquire about experiences during the treatment process, including the responses of health professionals and the treatment setting, specifically targeting perceptions of the treatment setting (e.g., whether there were any perceptions that the setting or treatment was not gender inclusive, experiences about receiving treatment with youth of the opposite gender)

At the end of the review of the timeline/treatment experiences, the interviewer will inquire about plans for follow-up in the community, and perceptions (i.e., thoughts/feelings) about the continuing care available.

“Do you have any suggestions about how we can make treatment here in this program better or more comfortable for you?

Thank you for your time. We really appreciate your input and help.”

*End interview, turn off audio-recording*
